# Emergency Department waiting times in a tertiary children’s hospital in Israel: a retrospective cohort study

**DOI:** 10.1186/s13584-017-0184-x

**Published:** 2017-11-10

**Authors:** Oren Feldman, Raviv Allon, Ronit Leiba, Itai Shavit

**Affiliations:** 1 Pediatric Emergency Department, Rambam Health Care Campus, Haifa, Israel; 2 Rappaport Faculty of Medicine, Technion-Israel Institute of Technology, Haifa, Israel; 3 Quality of Care Unit, Rambam Health Care Campus, Haifa, Israel; 4 POB 274, 3080500 Haifa, Israel

**Keywords:** Emergency, Child, Waiting time, Ethnicity

## Abstract

**Background:**

The purpose of this study was to assess ethnic differences in Emergency Department (ED) waiting times between Jewish and Arab children in a tertiary childrens’ hospital in Israel.

**Methods:**

This was a retrospective cohort study of all children who were admitted to the pediatric ED of the largest hospital in northern Israel, between January 2011 and December 2015. Univariate and multivariate analyses were used to assess the strength of association between ethnicity category and waiting time. The following were tested as possible confounders: triage category, age, gender, time of arrival category. The effect of nurse-patient ethnic concordance was assessed.

**Results:**

Full data were available in 82,883 patients, 55,497 (67.0%) Jews and 27,386 (33.0%) Arabs. Jews and Arabs had a similar median waiting time of 38 min (interquartile range [IQR] 22–63 and IQR 21–61, respectively). Ethnicity was not associated with a change in waiting time (*p* = 0.36). Factors that most influenced shorter waiting time were triage category 1 (change in waiting time: −25.5%; 95% confidence interval [CI]: −29.3 to −21.7), or triage category 2 (change in waiting-time: −21.8%; 95% CI: -23.7 to −20.05). Factors that most influenced longer waiting time were patient arrival during the morning shift period (change in waiting time: 5.45%; 95% CI: 4.59 to 6.31), or during the evening shift period (change in waiting time: 4.46%; 95% CI: 3.62 to 5.29). Ethnic discordance between triage nurses and patients did not yield longer waiting times.

**Conclusion:**

In this large pediatric cohort, ethnic differences in ED waiting time were not found.

## Background

Emergency Department (ED) waiting time, the period of time between ED registration and time of initial physician assessment, is an accepted measure for the quality of in-hospital emergency care. Longer waiting times not only causing patients’ dissatisfaction with the care received but may also result in patient deterioration and suffering and in delays in diagnosis and treatment [1, 2]. Previous studies revealed that ethnicity could independently be associated with ED waiting times. These U.S. studies reported that Hispanic children and non-Hispanic black children had longer waiting times than non-Hispanic white children [3, 4].

The population of Israel includes two ethnic groups; Jews (majority group) and Arabs (minority group) [5]. To date, there have been no studies that have evaluated possible differences in waiting time between Jews and Arabs in this bi-ethnic state.

The aim of this study was to determine if there are ethnic differences in ED waiting time between Jewish and Arab children in a tertiary children’s hospital in Israel.

## Methods

### Study design

A retrospective single-center cohort study was conducted over a period of 5 years. Patients who were admitted to the pediatric ED between January 1, 2011, and December 31, 2015, were included in the study.

### Setting

Located in the city of Haifa, Rambam Health Care Campus (RHCC) is the only tertiary hospital in the north of the country, and serves as a referral center for 12 district hospitals. RHCC is a public hospital that serves a population of more than two million residents of northern Israel; 600,000 are children, two-thirds are Jews, one-third are Arabs [6].

### Triage

The ED nursing staff in this hospital uses the pediatric Canadian Triage Acuity Scale to triage patients [7]. The pediatric Canadian Triage Acuity Scale is a triage method that has been widely used in Canada and other countries as of 2001 [8–10]. Using this method, patients are *divided* by the ED triage nurse into five categories according to their medical condition; level 1 - patient requires immediate evaluation and care, level 2 - patient requires evaluation and care within 15 min, level 3 – patient requires evaluation and care within 30 min, level 4 – patient requires evaluation and care within 60 min, and level 5 – patient requires evaluation and care within 120 min [8–10]. The triage process includes a brief history of the chief complaint and past medical history, obtaining vital signs, assessing pain level, and treatment where applicable. Once triage is completed, the patient is referred for assessment and/or treatment by a physician, or returns to the waiting room. Afterwards, patients can be observed for few hours, can be discharged home, or can be admitted to a pediatric ward. The ED does not include a short-stay unit.

### Study outcome measure

The primary outcome measure for this study was the ED waiting time; the time elapsed from patient’s registration at the reception desk until completion of triage process by the triage nurse.

### Data collection

Data were extracted from the hospital’s electronic medical records (‘Prometheus’). This computerized system is a mandatory working tool for all physicians, nursing staff, social workers, and any ED healthcare personnel. Patient’s demographic data, including identification number and ethnicity, are obtained from the parents by the ED clerk and recorded in the system. The following variables were abstracted: ED waiting time, demographics (age, gender), time of arrival (07:00–15:00, 15:00–23:00, 23:00–07:00), triage category (1–5), and ethnicity category (Arab, Jew). For each patient, triage nurse ethnicity was also extracted to examine the effect of nurse-patient ethnic concordance on waiting time. Triage nurse ethnicity was identified by name.

The study was approved by the institutional review board.

### Statistical analysis

Data were analyzed with SPSS 21 version (SPSS-IBM, Chicago, IL).

Univariate and multivariate analyses were used to assess the strength of association between ethnicity category (Jew, Arab) and ED waiting time (the dependent variable). The following variables were tested as possible confounders: triage category, diagnosis (medical, trauma), age (≤ 1 year, > 1 year), gender (male, female), time of arrival category (07:00–15:00, 15:00–23:00, 23:00–07:00). The normality of the quantitative parameter (waiting time) was tested by the Kolmogorov-Smirnov test and this variable was not normally distributed. Waiting times were positively skewed and were normalized with a natural logarithm transformation before analysis. We estimated the predicted probabilities by means of change in waiting time. The non-parametric Mann-Whitney test was used to test for a difference in waiting times between the two ethnic groups.

## Results

During the 5-year study period, 83,609 patients were admitted to the pediatric ED. Missing data was found in 726 patients. Full data were available in 82,883 patients, 55,497 (67.0%) Jews and 27,386 (33.0%) Arabs. 56,175 (67.8%) patients had medical emergencies and 26,708 (32.2%) patients had trauma-related emergencies. Male/female ratio was 57.7/42.3.

Jews and Arabs had a median waiting time of 38 min, interquartile range [IQR] 22–63 min, and 38 min, IQR 21–61 min, respectively.

Table 1 presents a bivariate analysis of waiting times in minutes. Table 2 presents a multivariate regression model for the change in waiting time in the study population (*n* = 82,883). Ethnicity did not influence ED waiting times. Factors that most influenced shorter waiting times were triage category 1 (change in waiting time: −25.5%; 95% CI: -29.3 to −21.7), or triage category 2 (change in waiting time: −21.8%; 95% CI: -23.7 to −20.05). Factors that most influenced longer wait times were patient arrival during the morning (change in waiting time: 5.45%; 95% CI: 4.59 to 6.31), or during the evening (change in waiting time: 4.46%; 95% CI: 3.62 to 5.29).

**Table 1:** Bivariate analysis of ED waiting times (*n* = 82,883)

Predictors of ED waiting time^a^	Unadjusted waiting time in minutes (95% CI)
Age	
≤ 1 year > 1 year	37.94 [37.92 to 37.96] 38.25 [37.98 to 38.45]
Gender	
Male Female	37.48 [37.25 to 37.68] 38.10 [37.85 to 38.26]
Ethnicity	
Jewish Arab	38.31 [37.91 to 38.54] 37.95 [37.62 to 38.24]
Diagnosis	
Trauma Medical	38.14 [37.92 to 38.37] 37.87 [37.65 to 38.25]
24-h time of arrival	
07:00–15:00 15:00–23:00 23:00–07:00	40.20 [39.87 to 40.52] 39.84 [39.53 to 40.16] 37.23 [36.45 to 39.68]
Triage level^b^	
1 2 3 4 5	28.31 [26.86 to 29.73] 29.72 [28.99 to 30.38] 33.86 [33.31 to 34.37] 35.30 [34.74 to 35.83] 42.45 [40.23 to 44.69]

Notes

*ED* Emergency Department, *CI* Confidence Interval

^a^ED waiting time = Time from patient registration at the reception desk until completion of triage process

^b^Pediatric Canadian Triage Acuity Scale:

Level 1- patient requires immediate evaluation and care;

Level 2 - patient requires evaluation and care within 15 min;

Level 3 - patient requires evaluation and care within 30 min;

Level 4 - patient requires evaluation and care within 60 min;

Level 5 - patient requires evaluation and care within 120 min

**Table 2:** Predictors of waiting time in the ED (*n* = 82,883)

Predictors of ED waiting time^a^	Unadjusted percentage of change in waiting time (95% CI)	*P* value	Adjusted percentage of change in waiting time (95% CI)	*P* value
Age				
≤ 1 year > 1 year	−0.15 [−0.20 to −0.11] Reference	<0.001	−0·18 [−0.23 to −0.13] Reference	< 0.001
Gender				
Male Female	−1.38 [−1.96 to −0·82] Reference	0.12	−1.25 [−1.79 to −0.65] Reference	0.25
Ethnicity				
Jewish Arab	0.83 [−0.23 to 1.43] Reference	0.46	0.28 [−0.32 to 0.87] Reference	0.36
Diagnosis				
Trauma Medical	0.39 [−0.21 to 0.99] Reference	0.20	0.02 [−0.51 to 0.73] Reference	0.68
24-h time of arrival				
07:00–15:00 15:00–23:00 23:00–07:00	5.79 [4.93 to 6.65] 4.86 [4.03 to 5.69] Reference	<0.001	5.45 [4.59 to 6.31] 4.46 [3.62 to 5.29] Reference	< 0.001
Triage level^b^				
1 2 3 4 5	−25.5 [−29.3 to −21.7] −21.8 [−23.7 to −20.05] −10.9 [−12.34 to −9.54] −7.1 [−8.57 to −5.71] Reference	<0.001	−25.5 [−29.03 to −21.5] −21.5 [−23.3 to −19.7] −10·8 [−12.22 to −9.41] −6.9 [−8.42 to −5.55] Reference	<0.001

Notes

*ED* Emergency Department, *CI* Confidence Interval

^a^ED waiting time = Time from patient registration at the reception desk until completion of triage process

^b^Pediatric Canadian Triage Acuity Scale:

Level 1- patient requires immediate evaluation and care;

Level 2 - patient requires evaluation and care within 15 min;

Level 3 - patient requires evaluation and care within 30 min;

Level 4 - patient requires evaluation and care within 60 min;

Level 5 - patient requires evaluation and care within 120 min

### Waiting times and nurse-patient ethnic concordance

Overall, 81,252 (98%) patients were triaged by 12 Jewish nurses and five Arab nurses. Triage nurse identity was missing in 1631 (2%) patients. A total of 57,698 (71%) patients were triaged by Jewish nurses and 23,544 (29%) were triaged by Arab nurses (Table 3).

**Table 3:** Nurse-patient ethnic concordance. Data are waiting times, median (interquartile range), minutes

	Jewish nurse	Arab nurse
Jewish patient	38 (22–64)	38 (22–60)
Arab patient	38 (22–67)	38 (24–67)

## Discussion

Previous studies have shown that ED delay can be associated with adverse outcomes in children. Bonadio et al. reported that in children, increasing the period of time from ED presentation to appendectomy was associated with an elevated risk of developing appendix perforation. Depinet et al. reported an association between ED crowding and delays in the reassessment of critically abnormal vital signs in children [1, 2]. Kennebeck et al. showed that crowding and ED delay were associated with delays in the receipt of antibiotics for febrile neonates [11].

In our large cohort, a multivariate regression analysis showed that ED waiting time was not influenced by ethnicity, and that the two ethnic groups had a similar median waiting time of 38 min. These findings suggest that ethnicity did not play a role in determining waiting time in this ED. Our findings differ from some United States studies in which ethnic/race disparity in ED waiting times was reported in adult and pediatric EDs [3, 4]. These studies found significant differences in ED waiting times between various ethnic groups, including non-Hispanic whites, non-Hispanic blacks, and Hispanic whites. Potential explanations for this phenomenon were language barrier, discrimination and sociocultural factors. Provider-related variables, such as prejudice and stereotyping, were also suggested as possible causes [3, 4, 12, 13]. In Israel, due to the National Health Insurance Law which came into effect in 1995, a patient admitted to the ED of a public hospital is covered by universal health insurance and is not required to pay for any treatment [14]. The fact that health care is covered equally for Jews and Arabs may have contributed to the finding of similar ED waiting times between the two ethnic groups.

Our study is the first to assess ED waiting times in a bi-ethnic population of Jews and Arabs. An important finding of our study is that ethnic discordance between nurses and patients did not yield longer waiting times (Table 3). This finding suggests that triage nurses in this department do not discriminate between ethnicities. A possible explanation could be the fact that triage nurses in this department undergo regular in-hospital triage training, to ensure that all children are assessed and prioritized for care based on clinical need. Nurse training includes educational sessions that contain simultaneous multiple patient simulations, human patient simulations, and online learning courses throughout the year [15, 16].

Our study has limitations inherent in a retrospective study, including dependence on the quality of the database. We believe that this factor had minimal impact because of the high quality of the hospital electronic registry. This computerized system consists of in-house Oracle based software (Prometheus) which was specially designed for storing data in real time. A second limitation is that this study is a single center study; therefore, conclusions may not apply to other EDs in Israel.

In conclusion, we found that waiting times were not influenced by ethnicity, were similar for both ethnic groups, and that triage nurses did not discriminate between the two ethnic groups. These data suggest that there was no ethnic disparity with regard to ED waiting time.

Our data provide evidence for equity in emergency care between Jews and Arabs in this ED. Further research on a national level is needed to clarify if the findings of this study apply to other EDs in Israel.

## Abbreviations

ED: Emergency Department
